# The Role of Type 2 Diabetes in Pancreatic Cancer

**DOI:** 10.7759/cureus.26288

**Published:** 2022-06-24

**Authors:** Sheeba George, Wilford Jean-Baptiste, Amina Yusuf Ali, Bithaiah Inyang, Feeba Sam Koshy, Kitty George, Prakar Poudel, Roopa Chalasani, Mastiyage R Goonathilake, Sara Waqar, Lubna Mohammed

**Affiliations:** 1 Research, California Institute of Behavioral Neurosciences & Psychology, Fairfield, USA; 2 Pediatrics, California Institute of Behavioral Neurosciences & Psychology, Fairfield, USA; 3 Internal Medicine, Chitwan Medical College of Medical Science, Chitwan, NPL; 4 Pediatrics/Internal Medicine, California Institute of Behavioral Neurosciences & Psychology, Fairfield, USA; 5 Internal Medicine, California Institute of Behavioral Neurosciences & Psychology, Fairfield, USA

**Keywords:** antidiabetic agents, pancreatic ductal adenocarcinoma, pancreatic neoplasm, diabetes mellitus type 2, type 2 diabetes

## Abstract

The incidence of type 2 diabetes mellitus (T2DM) and its potential complications, such as cancers, are increasing worldwide at an astounding rate. There are many factors such as obesity, diabetes, alcohol consumption, and the adoption of sedentary lifestyles that are driving pancreatic cancer (PC) to become one of the leading causes of cancer mortality in the United States. PC is notorious for its generic symptoms and late-stage presentation with rapid metastasis. The connection between T2DM and the risk of PC development is multifaceted and complex. Some of the proposed theories reveal that chronic inflammation, insulin resistance, hyperinsulinemia, hyperglycemia, and abnormalities in the insulin and insulin-like growth factor axis (IGF) contribute to the disease association between these two conditions. This literature review aims to highlight relevant studies and explore the molecular mechanisms involved in the etiology of diabetes and its impact on PC development, as well as the role of anti-diabetic agents on PC. Despite extensive studies, the exact interaction between T2DM and PC remains obscure and will need further investigation. According to current knowledge, there is a substantial link between diabetes, obesity, and dietary patterns in the development and progression of PC. Consequently, focusing our efforts on preventive measures by reducing modifiable risk factors remains the most effective strategy to reduce the risk of PC at this time. Antidiabetic drugs can have various effects on the occurrence and prognosis of PC with metformin offering a clear benefit of inhibiting PC and insulin increasing the risk of PC. The development of future novel therapies will require a deeper knowledge of the triggering mechanisms and interplay between these two disease states.

## Introduction and background

Pancreatic cancer (PC) is an aggressive malignancy with an overall poor prognosis [1]. Pancreatic ductal adenocarcinoma (PDAC) is the most common and fatal form of PC, accounting for more than 90% of pancreatic malignancies [1]. The latest data obtained from National Cancer Institute’s Surveillance, Epidemiology, and End Results Program (SEER) database showed the relative five-year survival rate for people with PDAC to be 11.5% [2]. PC is the fourth leading cause of cancer death in the United States, and based on the current rising trends, it is projected that by 2030, PC will become the second leading cause of cancer-related mortality in the United States [3,4]. According to the American Cancer Society, it is estimated that in 2022, about 62,210 people, consisting of 32,970 males and 29,240 females, will be diagnosed with PC, and about 49,830 people will die from it in the United States this year [5]. It is considered highly aggressive as it can metastasize early in the disease course, making it resistant to treatment modalities [6]. Advanced patient age, retroperitoneal tumor location, generic symptoms, and lack of screening tools further limit the treatment options for this deadly disease [6].

The American Diabetic Association (ADA) defines diabetes mellitus (DM) as a group of metabolic diseases characterized by chronic hyperglycemia resulting from impaired metabolism of carbohydrates, fats, and proteins [7]. Type 2 diabetes mellitus (T2DM), also known as non-insulin-dependent diabetes mellitus (NIDDM), is caused by insulin resistance (IR), and it is the most prevalent type, accounting for about 90-95% of the cases [7]. It is also associated with a higher body mass index (BMI), making it an independent risk factor for PDAC. Obesity and T2DM act synergistically to induce the development of PC [8]. Obese patients have a higher degree of IR, thereby making them prone to T2DM [8]. The International Diabetic Federation (IDF) estimates that approximately 537 million adults live with diabetes worldwide, and it is projected to increase to 643 million by 2030 and 783 million by 2045 [9].

**Figure 1:**
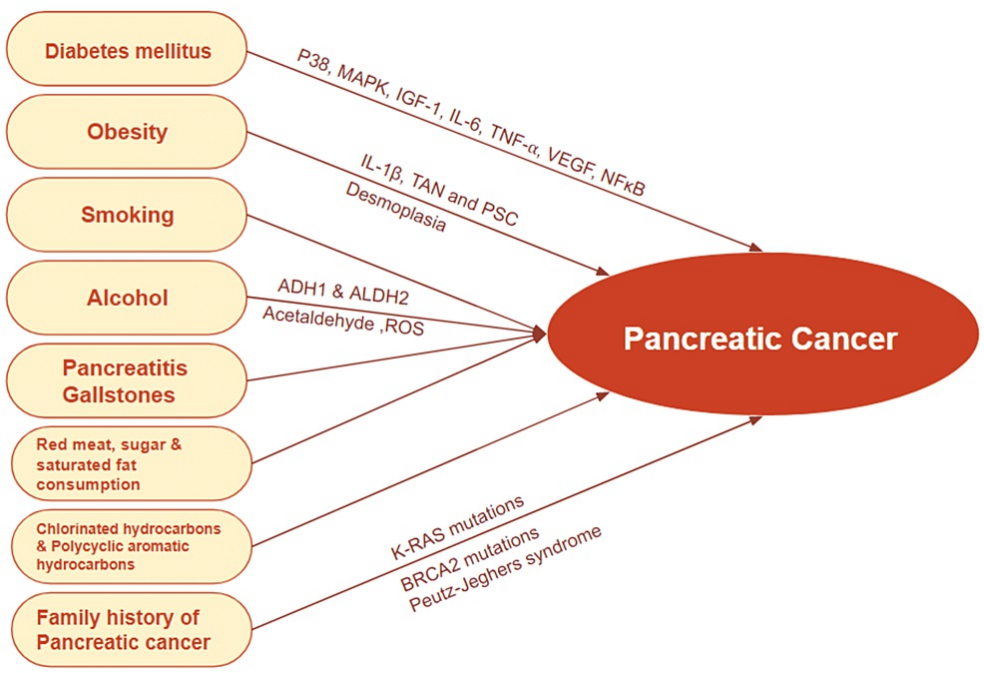
A schematic representation of some of the common risk factors for pancreatic cancer. Image created using Google Slides [13]. MAPK: mitogen-activated protein kinase; IGF-1: insulin-like growth factor 1; IL-6: interleukin-6; TNF-α: tumor necrosis factor-alpha; VEGF: vascular endothelial growth factor; NF-κB: nuclear factor kappa B; IL-1β: interleukin-1 beta; TAN: tumor-associated neutrophil; PSC: pancreatic stellate cells; ADH1: alcohol dehydrogenase 1; ALDH2: alcohol dehydrogenase 2; ROS: reactive oxygen species, K-RAS: Kirsten rat sarcoma virus gene; BRCA2: breast cancer gene 2

Diabetic patients have a two-fold risk of developing various types of cancer, such as colorectal, endometrial, renal, breast, liver, and pancreatic [10]. Multiple studies have shown a complex and bidirectional relationship between T2DM and PDAC [11]. Most common risk factors for PC include, but may not be limited to, family history, genetic mutations, cigarette smoking, sedentary lifestyle, obesity, DM, and chronic pancreatitis [12]. Smoking is a known potent carcinogen for PC. Other factors such as alcohol abuse, pancreatitis, and exposure to chemical carcinogens could also contribute to the development of PC [13]. Currently, there is a lack of diagnostic and therapeutic tools to detect and treat PDAC effectively; therefore, it is paramount to focus on prevention. This can be done by identifying modifiable risk factors that promote the underlying molecular mechanism in PDAC and investigating methods to defuse those risk factors [8]. A representation of some of the common risk factors for PC has been illustrated in Figure 1.

Search strategy

A detailed search was carried out for relevant articles in PubMed and PMC database using keywords and combinations such as “diabetes”; “pancreatic cancer” and “diabetes”; “pancreatic ductal adenocarcinoma” and “diabetes”; “antidiabetic agents” and “pancreatic cancer” and “diabetes.” The references of the pertinent articles were also searched for additional appropriate studies. All the articles taken into consideration were chosen without restriction at the time of publication or study type, that is, case-control and cohort studies, clinical trials, meta-analysis, traditional reviews, and systematic reviews. No demographic limitations were included in the search. Animal studies were excluded. The search was restricted to only English-language literature.

## Review

Diabetes, a risk factor for pancreatic cancer

Diabetes can be classified into three main subtypes based on its metabolic and hormonal features [14]. Type 1 diabetes mellitus (T1DM) is characterized by the absolute deficiency of endogenous insulin and requires exogenous insulin for survival. T2DM is associated with IR in peripheral tissues and is characterized by hyperglycemia and hyperinsulinemia [14]. Type 3c (secondary) diabetes (T3cDM) or pancreatogenic diabetes is an emerging subtype, commonly seen in conditions that damage the pancreas, such as chronic pancreatitis (80%), cystic fibrosis, pancreatic surgery, and hemochromatosis, and it is often characterized by severe deficiency of all pancreatic hormones involved in glucose homeostasis [15]. Long-standing T2DM is a significant risk factor for multiple types of malignancies, including PC, and several studies have shown that approximately 85% of patients had concurrent diabetes at the time of PC diagnosis [16]. Two separate studies by Batty et al. and Jeet et al. showed a direct correlation between fasting glucose and the risk of developing PC [17,18]. A study in Finland by Stolzenberg-Solomon et al. involving 29,133 smokers who were followed over the course of 10 years concluded that long-standing impairments from diabetes were necessary for PC development. Specifically, in this study, the hazard ratio did not increase significantly until the 10-year follow-up mark for PC [19]. In a study, Yacoub et al. showed that long-term, mostly, T2DM patients had about a two-fold increased risk of developing PC [20]. Another meta-analysis of 35 cohort studies established a correlation between DM and PC, independent of other confounding factors such as sex, geographical location, smoking status, and alcohol use or body mass index [21]. Approximately 50% of PC patients were noted to have DM at the time of diagnosis, out of which the majority were diagnosed with new-onset diabetes two to three years before the diagnosis of PC. This new-onset diabetes, secondary to other causes, is also known as T3CDM [22]. Although the association between DM and PC has been extensively studied, the relationship between the two is yet to be fully understood due to difficulties distinguishing T2DM from T3Cdm [22]. Lowenfels et al., in their study, showed that patients with chronic pancreatitis have an increased risk of developing PC [23]. Meta-analyses performed on large studies involving both case and cohort studies have consistently shown a two-fold increased risk of PC in diabetes patients compared to those without, with a stronger association existing in cohort studies compared to case-control studies [24-26].

Potential mechanisms of diabetes on the development of pancreatic cancer

Chronic inflammation, hyperglycemia, hyperinsulinemia, and abnormalities in insulin/insulin-like growth factor 1 (IGF-1) pathways are some of the proposed molecular mechanisms by which DM and PC are closely related, with emerging evidence showing a positive association between DM and the risk of PC [22,27,28].

Insulin Resistance, Hyperinsulinemia, and Pancreatic Cancer

IR is the hallmark of T2DM. Higher insulin levels are responsible for stimulating the insulin/IGF-1R signaling pathway via serine phosphorylation of insulin receptor substrates (IRS), which, in turn, leads to the activation of the phosphatidyl inositol-3 kinase and mammalian target of rapamycin (PI3K/mTOR) pathway [29]. Frasca et al. and Kornmann et al. in their studies suggested that IGFR-1 and chronic hyperinsulinemia play a vital role in favoring cancer initiation, malignant transformation, and metastasis of various types of cancer in patients with diabetes [30,31]. Consequently, hyperinsulinemia and subsequent rise in IGF-1 contribute to the development and progression of PC by promoting cellular proliferation and differentiation, inhibiting apoptosis, and enhancing angiogenesis [32]. PC cells are also known to express insulin and IGF-1 receptors and IRS-1,2 in large amounts, further suggesting an essential role of the insulin/IGF-1R pathway in developing PC [31]. Similarly, inhibition of IGF-1R showed an improved response of colon cancer stem cells to chemotherapy [33]. However, the signaling pathways activated by insulin/IGF-1 receptors in PC have not been fully elucidated, and thus further studies are required to gain a better understanding of molecular mechanisms involved in the development and progression of PC.

The P38 signaling transduction pathway is a class of mitogen-activated protein kinases (MAPKs) that plays a crucial role in many cellular processes, such as cell differentiation, inflammation, proliferation, and cell death [34]. It is activated in response to high glucose levels, stressful stimuli, and inflammation and is responsible for the rapid proliferation and invasion of PC cells [34,35]. DM and its concurrent chronic inflammatory processes lead to tumor progression, epithelial‐mesenchymal transition (EMT), and metastasis by the production of inflammatory cytokines (interleukin 6 (IL‐6) and tumor necrosis factor-alpha (TNFα)), and leading to the activation of kinases p38 MAPK and nuclear factor kappa B (NFкB) [35]. NFкB is a critical transcription factor that plays a vital role in cellular proliferation, morphogenesis, and apoptosis and promotes inflammation by producing inflammatory cytokines (e.g., IL‐6, IL‐8, IL‐1β, and TNFα), chemokines, and adhesion molecules triggering PC cell growth [36].

High insulin levels decrease the hepatic production of IGF‐binding proteins (IGFBPs), thereby increasing levels of IGF-1. Many studies have shown a directly proportional relationship between the concentration of IGF-1 and the rate of PC proliferation [37,38]. Type 2 diabetics with elevated insulin and IGF-1 were noted to have larger pancreatic tumors when compared to normoglycemic controls [39]. Insulin possesses metabolic and mitogenic effects by promoting glucose transport and potentiating cellular mitogenic responsiveness to growth factors, respectively [40]. Cancer cells thrive on glucose, thus confirming that hyperglycemia accelerates PC development. It also enhances PC proliferation by the activation of epidermal growth factor (EGF) and increases the expression of pluripotency stem cell markers (e.g., SOX2, OCT4, and Nanog) by activating transforming growth factor-beta 1 (TGF-B1) [41,42]. Additional studies are required to better understand the molecular mechanisms involved in chronic metabolic diseases and PC stem cells.

Role of Inflammation and Pancreatic Cancer

The persistent state of IR with hyperglycemia and hyperinsulinemia, along with the proinflammatory state, causes the slow deterioration of pancreatic beta-cell function, thereby increasing the risk of developing PC [43]. Evidence supports the involvement of inflammation in the pathogenesis of T2DM and PC development.

NFкB and signal transducer and activator of transcription 3 (STAT3) pathways function closely and similarly as promoters of cell proliferation and inhibitors of programmed cell death [28]. In addition, TGF-B1 decreases the expression of E-cadherin (a tumor suppressor), which results in the induction of EMT. The tumor microenvironment (TME) is the environment surrounding the tumor constituting immune cells, endothelial cells, and cancer-associated fibroblasts (CAFs), which play a crucial role in PC invasiveness and metastasis [28].

During inflammation, immune cells release many cytokines into the microenvironment resulting in PC growth and development [44-47]. Receptor for advanced glycation end-products (RAGE) plays an essential role in contributing to a sustained inflammatory state in patients with T2DM. Chronic stress and hyperglycemia lead to increased production of advanced glycation end-products (AGE), which activates RAGE and Kirsten rat sarcoma (Kras). RAGE binds typically to the S-100 protein family, which plays a significant role in inflammation and cancer development, including PC. Thus, the excessive activation of RAGE may be a contributing factor to the development of PDAC in type 2 diabetic patients [48-50]. Obesity works synergistically with T2DM in increasing the risk of PC. The inflammatory pathway through obesity is thought to propagate a favorable microenvironment for tumor growth. Furthermore, studies have shown a strong association between visceral adiposity and gastrointestinal cancers including PC [51].

Nutrition and Pancreatic Cancer

According to research, the pro-inflammatory dietary pattern may exacerbate the risk of type 2 diabetes [52]. The Western diet, constituting high sugar, high fat, and refined carbohydrates, worsens inflammation by increasing the production of reactive oxygen species (ROS) and by the activation of NFкB and activator protein 1, which are the key transcription factors involved in genomic derangement and tumor development [53-55]. In contrast, the Mediterranean diet, which includes whole grains, fruits, vegetables, seafood, beans, and nuts, was associated with decreased inflammatory markers and decreased PC risk [56-58].

A diet consisting of high calories and poor nutritious value leads to excessive inflammation by producing inflammatory cytokines (e.g., IL‐6, IL‐8, IL‐1β, TNF‐α, and IFN‐ϒ), advancing to higher production of ROS. Therefore, it is proposed that high ROS production may exacerbate DNA mutations and PC progression [59].

Genetic Mutations Driving Pancreatic Cancer

**Figure 2:**
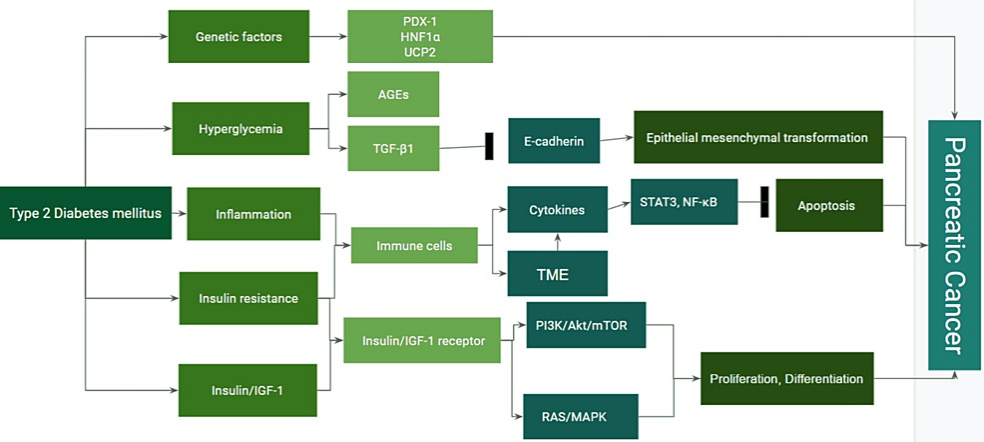
Mechanisms involved between type 2 diabetes mellitus and pancreatic cancer. Created using Google Slides by Sara Waqar [28]. AGEs: advanced glycation end products; AMPK: adenosine monophosphate protein-activated kinase; IGF-1: insulin-like growth factor-1; LKB: liver kinase B; MAPK: mitogen-activated protein kinase; mTOR: mammalian target of rapamycin; NF-κB: nuclear factor kappa B; PI3K: phosphatidyl inositol-3 kinase; STAT3: signal transducer and activator of transcription 3; TGF-β1: transforming growth factor-β1; TME: tumor microenvironment; PDX1: pancreatic and duodenal homeobox-1; HNF1A: hepatocyte nuclear factor-1 alpha; UCP2: uncoupling protein 2

Certain pancreatic developmental genes such as *nuclear receptor subfamily 5 group A member 2* (*NR5A2*), *pancreatic and duodenal homeobox 1* (*PDX1*), and *hepatocyte nuclear factor-1 alpha* (*HNF1A*) have been identified as susceptibility factors for PC in T2DM patients. Monogenic diabetes, a rare form of diabetes seen in young people (types 4 and 5), is associated with heterozygous mutations in some genes, such as *PDX1 *and *HNF1A*. Variants of *PDX1 *and *HNF1A *genes are also linked with an increased risk of obesity, T2DM, or hyperglycemia [60-62]. Figure 2 summarizes the different mechanisms that are involved between T2DM and the development of PC [28].

Antihyperglycemic agents and pancreatic cancer

T2DM is treated with various pharmacological therapies such as metformin, sulfonylureas, thiazolidinediones (pioglitazone), sodium-glucose cotransporter 2 inhibitors (dapagliflozin), and insulin. A plethora of studies has shown that exogenous insulin can potentially increase the risk of PC. A case-control study by Bonny et al. involving 244 patients with PC and 459 controls was conducted to understand the effect of insulin versus non-insulin therapy on PC development. On investigation, it was found that the risk of developing PC was increased 6.49-fold in those treated with insulin versus 2.12-fold in those treated with oral hypoglycemic agents [63].

Metformin (1,1-dimethyl biguanide hydrochloride) has been one of the most widely used oral hypoglycemic agents in the treatment of T2DM since the 1950s. It decreases hepatic gluconeogenesis and increases peripheral glucose uptake in target tissues resulting in an overall decreased concentration of blood glucose [13] It also decreases insulin levels by improving insulin sensitivity and minimizes the risk of developing PC [13]. Although it is a well-tolerated drug, it is generally contraindicated in patients with poor renal function due to the risk of lactic acidosis (<1/10,000).

Multiple studies indicate that metformin decreases the risk of PC by activating liver kinase B1 (LKB1)-adenosine monophosphate protein-activated kinase (AMPK) pathway, inhibiting hepatic gluconeogenesis and cancer cell proliferation [28]. The LKB1-AMPK pathway is a potent inhibitor of mTOR, leading to rapid inhibition of cellular protein synthesis and growth [28]. Metformin also disrupts NF-κB and hypoxia-inducible factor 1-alpha (HIF-1α), resulting in the inhibition of vascular endothelial growth factor (VEGF) and inflammatory cytokines such as IL-1, IL-6, and TNF‐α [64,65]. In 2011, Sadeghi et al. conducted a retrospective cohort study involving 302 patients to investigate the survival benefits of metformin in pancreatic neoplasm and DM. The results showed a longer median survival rate in those who received metformin therapy versus non-users: 16.6 versus 11.5 months, respectively. The risk of death was also decreased by 33% in those who received metformin compared to those who did not [66].

In addition, metformin has been proven to be an effective anti-neoplastic drug in patients with diabetic colon cancer and breast cancer [67,68]. A large case-control study to investigate the effect of metformin in PC was conducted at M.D Anderson Cancer Center. The study included 973 PC patients, of whom 259 were diabetic. The results showed that diabetic patients treated with metformin had a lower risk of developing PC compared to those who did not receive metformin, whereas diabetic patients who received insulin therapy had a higher risk of PC [69]. Incretin-based therapies glucagon-like peptide-1 (GLP1) analogs, and inhibitors of dipeptidyl peptidase-IV, the enzyme that metabolizes GLP1, have shown an association with acute pancreatitis [8]. Even though the clinical significance is uncertain, the long-term effects are being investigated [8]. In addition to the anti-hyperglycemic effects of metformin, its role as an effective anti-neoplastic drug is promising as it can bring forth exciting clinical applications in the treatment outcome of patients with this deadly disease. Further studies on other anti-diabetic agents need to be conducted to clarify the association of its effect on pancreatic cancer.

## Conclusions

PC remains a deadly disease, with studies suggesting an increased risk of T2DM individuals developing PC. Even though treatments such as chemotherapy and immunotherapy have increased the overall median survival, the truth remains that the total survival in these cases remains lower than a year. The interplay between how T2DM triggers PC remains a subject of future studies. Current understanding has shown a strong correlation between DM, obesity, and dietary patterns in the development and progression of PC. Therefore, focusing our shift on reducing the modifiable risk factors remains the most effective option to minimize the risk of PC. The effects of antidiabetic agents on the occurrence and prognosis of PC have been studied extensively. The mechanism by which metformin decreases PC risk and insulin exerts a converse effect has been analyzed. Through further investigations, if metformin is found to have an antitumor effect, it would be a breakthrough in improving PC prognosis. A further understanding of the triggering mechanism and interplay between the different biomarkers is key to developing future novel therapies.
